# Therapeutic Schedule Evaluation for Brain-Metastasized Non-Small Cell Lung Cancer with A Probabilistic Linguistic ELECTRE II Method

**DOI:** 10.3390/ijerph15091799

**Published:** 2018-08-21

**Authors:** Ling Pan, Peijia Ren, Zeshui Xu

**Affiliations:** 1 Business School, Sichuan University, Chengdu 610064, China; 2017225020008@stu.scu.edu.cn (L.P.); 2016325020009@stu.scu.edu.cn (P.R.); 2 School of Computer and Software, Nanjing University of Information Science and Technology, Nanjing 210044, China

**Keywords:** brain-metastasized NSCLC, therapeutic schedules, indicator evaluation system, probabilistic linguistic term set, ELECTRE II method

## Abstract

With the rapid development of modern medicine, therapeutic schedules of brain-metastasized non-small cell lung cancer (NSCLC) are expanding. To assist a patient who suffers from brain-metastasized NSCLC to select the most suitable therapeutic schedule, firstly, we establish an indicator system for evaluating the therapeutic schedules; then, we propose a probabilistic linguistic ELECTRE II method to handle the corresponding evaluation problem for the following reasons: (1) probabilistic linguistic information is effective to depict the uncertainty of the therapeutic process and the fuzziness of an expert’s cognition; (2) the ELECTRE II method can deal with evaluation indicators that do not meet a fully compensatory relationship. Simulation tests on the parameters in the proposed method are provided to discuss their impacts on the final rankings. Furthermore, we apply the proposed method to help a patient with brain-metastasized NSCLC at the Sichuan Cancer Hospital and Institute to choose the optimal therapeutic schedule, and we present some sensitive analyses and comparative analyses to demonstrate the stability and applicability of the proposed method.

## 1. Introduction

The incidence of lung cancer continues to increase all over the world. According to the latest data released by the National Cancer Center, compared with the other cancers, the incidence and the mortality of lung cancer both are number one in China. Meanwhile, with the aggravation of the population aging process and the degradation of the environment, the incidence of lung cancer is growing at 27% per year in China. Non-small cell lung cancer (NSCLC), which takes up 80% of lung cancer [1], threatens human health nowadays. In 44% of patients with NSCLC there occur brain metastases [2], and the 5-year survival rate of brain-metastasized NSCLC is very low. Under such circumstances, many medical researchers and workers have endeavored to investigate it. Some modern therapeutic schedules for brain-metastasized NSCLC have been emerging, such as Epidermal Growth Factor Receptor inhibitor therapy [3,4], Anaplastic Lymphoma kinase (ALK) inhibitor therapy [5,6], and immunotherapy [7,8].

The expansion of therapeutic schedules gives a new prospect for the success of conquering brain-metastasized NSCLC. However, for a patient, it is hard to find an optimal choice in a series of therapeutic schedules since the prognostic projection of each patient may be different [9]. Current research on therapeutic schedules is mostly based on the diagnosed patients’ clinical follow-up by some statistical techniques, which means that the results are obtained from the average perspective. However, patients may be more concerned about their own survival gains from each therapeutic schedule compared to the average or median survival gains. Therefore, the idea about determining the therapeutic schedule for brain-metastasized NSCLC in an individualized way was proposed [10].

Actually, the optimal therapeutic schedule for a patient is usually selected by expert consultation among several possible schedules. For the characteristics of medical events, the experts tend to use linguistic variables [11] rather than crisp numbers to express their judgments for some indicators. For instance, the terms that the experts commonly use to describe an evaluation of the response to a therapeutic schedule are complete response (CR), partial response (PR), stable disease (SD), and progressive disease (PD) [12]. During the process to choose an optimal therapeutic schedule for any individual brain-metastasized NSCLC patient, the experts face uncertainty about the success of the therapeutic schedule due to their limitations on access to information and inability to evaluate the therapeutic effect on any individual patient. In this situation, the traditional linguistic variables which only use single or simple linguistic terms to describe the assessment information are inefficient. For example, by combining the therapeutic effects of a patient on response, efficiency, and any other aspects, an expert may think that it is “between average and good”. Hesitant fuzzy linguistic term sets (HFLTSs) [13], which contain several consecutive linguistic terms, were proposed to reflect this kind of evaluation.

However, the existing descriptive techniques are insufficient in some cases. For example, during the therapeutic schedule response evaluation process, an expert may think that the response is “PR” with the probability 40% and is “SD” with the probability 60%. What is more, in the event of expert consultation, which focuses on evaluating the response of a therapeutic schedule for a patient, 40% of experts think that the response is “PR”, 20% of experts think that it is “SD”, and the remaining 40% of experts choose to abstain. To increase the elasticity of linguistic terms to portray such more general situations, Pang et al. [14] proposed a probabilistic linguistic term set (PLTS) by giving each possible linguistic term a corresponding probability to denote its weight or importance degree. Considering that it can depict the fuzziness in a novel way, which addresses the uncertainties and complexities existing in medical issues, we utilize it to represent the evaluation information of therapeutic schedules in this paper.

A further difficulty of the therapeutic schedule evaluation problem for brain-metastasized NSCLC is to find a suitable method. Currently, there exists some research related to the selection of a therapeutic schedule. From the perspective of statistics, the efficiency of different classical therapeutic schedules for patients who have undergone resection for brain metastases has been researched by analyzing retrospectively the clinical trial data [1,15]. From the perspective of pharmacoeconomics, the advanced therapeutic schedules have been proven to be superior to the classical therapeutic schedules at cost-utility based on the randomized clinical trial data [16,17]. From the perspective of drug resistance, the efficiency of combination therapies has been evaluated by using population and cellular-scale clinical data [18,19]. However, the existing studies have the following defects:

(1) The existing methods mostly focus on evaluating the therapeutic schedules based on historical clinical trial data, and they fail to provide an exact solution for a patient to choose the optimal therapeutic schedule;
(2) The current mainstream methods only consider one or two attributes, such as the response and cost of schedule. Actually, choosing an optimal therapeutic schedule for any individual patient should be considered as a complex multi-attribute decision making (MADM) problem since it needs to consider a lot of indicators, such as the response of schedule, the applicability of the schedule, and the evidence of the experts’ evaluations, which have not yet been addressed;
(3) The evaluation values in the current methods are expressed in crisp numbers, which cannot describe some indicators, such as the quality of life. Furthermore, crisp numbers are insufficient to represent the uncertainties and complexities existing in the therapeutic schedule selection process and the fuzziness of the experts;
(4) The current methods just take the average values of all evaluation values on indicators as the results, which include an assumption that the relationship between the indicators is fully compensatory. Obviously, this assumption is irrational in therapeutic schedule selection problems for brain-metastasized NSCLC.

The ELECTRE II method, which is a MADM method based on an outranking relation [20], can solve problems whose attributes’ relationship is partially compensatory. With such an advantage, it has been extended into different environments, such as fuzzy environment [21], intuitionistic fuzzy environment [22], hesitant fuzzy environment [23], linguistic environment [24], and interval 2-tuple linguistic environment [25]. However, these ELECTRE II methods cannot adequately portray the characteristics of expert consultation and situations with lots of uncertainties.

To this end, this paper proposes the PLTS-based ELECTRE (PL-ELECTRE) method to evaluate therapeutic schedules for brain-metastasized NSCLC. It applies probabilistic linguistic information to present evaluation opinions of experts, and utilizes the ELECTRE method to address the partially compensatory relationship between the attributes in MADM problems.

The rest of this paper is constructed as follows: Section 2 reviews some concepts related to PLTSs. In Section 3, an indicator system for therapeutic schedule evaluation is established. In Section 4, we define a probabilistic linguistic chi-square method used to derive the weight vector of the indicators, and then propose a PL-ELECTRE method based on the possibility degrees of PLTSs. Section 5 provides some simulation tests on the parameters in the PL-ELECTRE method to discuss their impacts on the final rankings. In Section 6, the proposed method is applied to evaluate five sample therapeutic schedules for a patient in Sichuan Cancer Hospital and Institute. Furthermore, sensitive analyses and comparative analyses are presented to manifest the rationality and stability of the proposed method. The paper finishes with conclusions in Section 7.

## 2. Preliminaries

A subscript-symmetric additive linguistic term set $S=\left\{s_{g}|g=-\tau ,\cdots ,-1,0,1,\cdots ,\tau \right\}$ [26] satisfies the following conditions: (1) The set is ordered: if g≥h, then $s_{g}\geq s_{h}$; (2) The negation operator is: $neg(s_{g})=s_{-g}$. To describe the hesitation and uncertainties of experts, the probabilistic linguistic term set (PLTS) [14] was proposed based on $S=\left\{s_{g}|g=-\tau ,\cdots ,-1,0,1,\cdots ,\tau \right\}$:

(1) $$L(p)=\left\{L^{\left(l\right)}(p^{\left(l\right)})|L^{\left(l\right)}\in S,p^{\left(l\right)}\geq 0,\sum_{l=1}^{\#L(p)}p^{\left(l\right)}\leq 1\right\}$$

where $L^{\left(l\right)}(p^{\left(l\right)})$ is a probabilistic linguistic element (PLE) that contains the *l*-th linguistic term and its corresponding probability $p^{\left(l\right)}$, and #L(p) is the number of linguistic terms in L(p). To improve its usability, the transformed PLTS was given as [14]:

(2) $$L{\left(p\right)}^{N}=\left\{L^{N(l)}(p^{N(l)})|L^{N(l)}=L^{\left(l\right)}\in S;p^{N(l)}\geq 0;\sum_{l=1}^{\#L(p)}p^{N(l)}=1\right\}$$

where $p^{N(l)}=p^{\left(l\right)}/\sum_{l=1}^{\#L(p)}p^{\left(l\right)}$.

Later on, a PLTS with ordered PLEs was introduced to conform to practice as follows [27]: for a PLTS $L(p)=\left\{L^{\left(l\right)}(p^{\left(l\right)})|\;l=1,2,\cdots ,\#L(p)\right\}$, where $L^{\left(1\right)}\left(p^{\left(1\right)}\right)$ and $L^{\left(2\right)}\left(p^{\left(2\right)}\right)$ are two elements of L(p), and $r^{\left(1\right)}$ and $r^{\left(2\right)}$ are the subscripts of the linguistic terms $L^{\left(1\right)}\left(p^{\left(1\right)}\right)$ and $L^{\left(2\right)}\left(p^{\left(2\right)}\right)$, we have

(1) When $r^{\left(1\right)}p^{\left(1\right)}\neq r^{\left(2\right)}p^{\left(2\right)}$, if $r^{\left(1\right)}p^{\left(1\right)}>r^{\left(2\right)}p^{\left(2\right)}$, then $L^{\left(1\right)}\left(p^{\left(1\right)}\right)>L^{\left(2\right)}\left(p^{\left(2\right)}\right)$;
(2) When $r^{\left(1\right)}p^{\left(1\right)}=r^{\left(2\right)}p^{\left(2\right)}$, then
  (a) If $r^{\left(1\right)}\neq r^{\left(2\right)}$, then $L^{\left(1\right)}\left(p^{\left(1\right)}\right)>L^{\left(2\right)}\left(p^{\left(2\right)}\right)$ when $r^{\left(1\right)}>r^{\left(2\right)}$;
  (b) If $r^{\left(1\right)}\;=\;r^{\left(2\right)}$, then $L^{\left(1\right)}\left(p^{\left(1\right)}\right)>L^{\left(2\right)}\left(p^{\left(2\right)}\right)$ when $p^{\left(1\right)}>p^{\left(2\right)}$.

Since different PLTSs usually possess different numbers of PLEs, the operations between them are difficult to realize. To solve this problem, based on the transformed PLTS, the following rule was proposed to normalize it [14]: Let $L{\left(p\right)}_{1}$ and $L{\left(p\right)}_{2}$ be any two transformed PLTSs. If $\#L{\left(p\right)}_{1}>\#L{\left(p\right)}_{2}$, then we add $\#L{\left(p\right)}_{1}-\#L{\left(p\right)}_{2}$ linguistic terms to $L{\left(p\right)}_{2}$, where the added linguistic terms are the smallest one in $L{\left(p\right)}_{2}$, and their probabilities are zero.

Considering that a preference relation is an efficient tool to depict experts’ opinions, the probabilistic linguistic preference relation (PLPR) [27] was defined as follows:

For a set of indicators $A=\left\{a_{j}|j=1,2,\cdots ,m\right\}$, a PLPR on $S=\left\{s_{g}|g=-\tau ,\cdots ,-1,0,1,\cdots ,\tau \right\}$ can be represented as $B={\left(L{\left(p\right)}_{ij}\right)}_{m\times m}\subset A\times A$, where $L{\left(p\right)}_{ij}$ is a PLTS, which manifests the preference degrees of the indicator $a_{i}$ over the indicator $a_{j}$. For all i,j=1,2,…,m, $L{\left(p\right)}_{ij}$ should satisfy:

(1) $p_{ij}^{\left(l\right)}=p_{ji}^{\left(l\right)}$, $L_{ij}^{\left(l\right)}=neg\left(L_{ji}^{\left(l\right)}\right)$, $L{\left(p\right)}_{ii}=\left\{s_{0}(1)\right\}=\left\{s_{0}\right\}$, $\#L{\left(p\right)}_{ij}=\#L{\left(p\right)}_{ji}$;
(2) $L_{ij}^{\left(l\right)}p_{ij}^{\left(l\right)}\leq L_{ij}^{\left(l+1\right)}p_{ij}^{\left(l+1\right)}$ for i≤j, $L_{ji}^{\left(l\right)}p_{ji}^{\left(l\right)}\geq L_{ji}^{\left(l+1\right)}p_{ji}^{\left(l+1\right)}$ for i≥j.

## 3. Establishment of the Indicator System

In individualized treatment, there usually exist several possible therapeutic schedules. The experts need to consult on an optimal schedule for each patient. With this background, it is necessary to establish an indicator system to evaluate each therapeutic schedule.

Currently, several international authorities have put forward different evaluation indicator systems for brain-metastasized NSCLC. The National Cancer Comprehensive Network (NCCN) created an evaluation indicator system including efficiency, safety, evidence quality, evidence consistency, and affordability based on an evidence module [28]. The American Society of Clinical Oncology (ASCO) established an indicator system with clinical benefit, toxicity, and cost based on a value framework [29]. The European Society of Medical Oncology (ESMO) developed the Magnitude of Clinical Benefit Scale (MCBS) to evaluate therapeutic schedules, which mainly contains overall survival, progression-free survival, objective response rate, quality of life, and toxicity [30].

Even though the NCCN, ASCO, and ESMO guidelines have proposed some evaluation indicators, they still have certain flaws in practical situations, i.e., both the NCCN and ASCO guidelines do not contain the indicator quality of life and the ESMO guidelines do not consider the cost of a therapeutic schedule, which all are important for brain-metastasized NSCLC patients. In the following, we comprehensively establish a new evaluation indicator system to remedy the above flaws based on evidence-based medicine, pharmacoeconomics, and clinical experience. According to the similarities and differences between these indicators, the indicator system is divided into three aspects: responses, applicability, and evidence.

### 3.1. Response

In this paper, the response indicators describe the performance of a therapeutic schedule in prolonging survival, disease control, and quality of life, which focus on not only the quantity of survival but also the quality of survival. The specific contents of the response indicators are:

**Efficacy.** Efficacy refers to the degree of prolonging survival and disease control for any therapeutic schedule. It is one of the most popular indicators to evaluate the therapeutic schedule for cancer. It originated from the NCCN guidelines.

**Quality of life.** Quality of life (QoL) is a multidimensional concept including body function appearance, mental state appearance, psychological activities, society function, health perception, and the corresponding symptoms of the disease. As an emerging indicator, QoL is widely used in the medical field, and is particularly significant for terminal cancer patients at the dying end-stage.

### 3.2. Applicability

Besides the performance of response, the experts also need to consider the applicability of the therapeutic schedules from the perspective of the patient’s performance status (PS) and his/her financial status. The specific indicators are introduced as follows:

**Safety.** Safety refers to the degree of the therapeutic schedule’s toxicity and side-effects and their interference with daily activities for the patient. Safety evaluation is the primary goal of the therapeutic schedule’s evaluation, and a safe therapeutic schedule is also the prerequisite for curing diseases and improving a patient’s health.

**Affordability.** The concept of affordability is based on pharmacoeconomics. It refers to the patient’s ability to afford the cost of therapy, which includes the drug cost, supportive care cost, infusion cost, toxicity monitoring cost, and the toxicity management cost. Actually, many patients give up a more effacious therapeutic schedule due to their inability to afford the economic costs.

### 3.3. Evidence

The evidence indicators are proposed based on the idea of evidence-based medicine, which are manifest by the experts’ evaluations for the therapeutic schedules. Evidence mainly refers to the clinical evidence, including etiology, diagnosis, prevention, treatment, rehabilitation, and prognosis. The specific evidence indicators are presented as follows:

**Quality.** Quality refers to the reliability of the evidence. Usually, clinical evidence from large-sample randomized controlled trials (RCTs) and systematic reviews or meta-analyses has high quality. Conversely, evidence from only case reports and clinical experience has low quality.

**Consistency.** Consistency refers to the consistency of the results from multiple studies. This indicator is created to avoid errors in the evidence. It ensures the authenticity and reliability of the evidence, and is one of the most important indicators we need to consider.

## 4. Methodology

To solve the MADM problem about therapeutic schedule selection for brain-metastasized NSCLC, this section firstly introduces the chi-square method to obtain the weight vector of attributes, and then proposes the PL-ELECTRE method to evaluate the therapeutic schedule.

### 4.1. Probabilistic Linguistic Chi-Square Method

Since it is difficult for experts to provide accurate and complete attribute weights because of their cognition limitations and the problems’ complexities, the experts are willing to give preference information between two objects, and deriving the weights from preference relations is a significant way to process decision-making problems. Considering that therapeutic schedule selection for brain-metastasized NSCLC patients can be effectively handled in a probabilistic linguistic environment and the chi-square method [31] is a simple way to obtain the weights, in the following, we develop a chi-square method based on PLPR to derive the attribute weights.

**Definition** **1.** *Let*$B={\left(L_{ij}(p_{ij})\right)}_{m\times m}$*be a PLPR and*$B^{N}={\left(L_{ij}(p_{ij}^{N})\right)}_{m\times m}$*be its normalized form, then*B*is referred to as an expectedly consistent PLPR if*$e_{ij}=e_{io}+e_{oj}$(i,j,o=1,2,⋯,m)*, where the expected numerical value*$e_{ij}$*of*$L{\left(p\right)}_{ij}$*can be calculated by*

(3) $$e_{ij}=\frac{\sum_{l=1}^{\#L\left(p\right)}p_{ij}^{\left(l\right)}\left(\frac{r_{ij}^{\left(l\right)}}{T}+0.5\right)}{\sum_{l=1}^{\#L}p_{ij}^{\left(l\right)}},\;\mathrm{for}\;\mathrm{any}\;i,j=1,2,\cdots ,m$$

*where*#L(p)*is the length of the PLTSs,*T*is the number of linguistic terms in the set*$S=\{s_{g}|g=-\tau ,$⋯,−1,0,1,⋯,τ}*, and*$p_{ij}^{\left(l\right)}$*and*$r_{ij}^{\left(l\right)}$*are respectively the probability and the subscript of the l*-th *linguistic term in*$L{\left(p\right)}_{ij}$*.*

For a set of attributes $A=\left\{a_{i}|i=1,2,\ldots ,m\right\}$, suppose that the experts give their preferences as $B={\left(L{\left(p\right)}_{ij}\right)}_{m\times m}\subset A\times A$, where B is a PLPR. Let the vector $w={\left(w_{1},w_{2},\cdots ,w_{m}\right)}^{T}$ be the attribute weights, where $w_{i}\geq 0$, $\sum_{i=1}^{n}w_{i}=1$, and i=1,2,⋯,m, then according to the property of expectedly consistent PLPR, we have

(4) $$e_{ij}=w_{i}-w_{j}+0.5,\;\mathrm{for}\;\mathrm{any}\;i,j=1,2,\cdots ,m$$

However, it is often difficult for the PLPR to satisfy completely consistent conditions in an actual decision-making environment. To depict the deviation between the PLPR and the corresponding consistent PLPR, the following function is introduced:

(5) $$F\left(w\right)=\sum_{i=1}^{m}\sum_{j=1}^{m}\frac{{\left(e_{ij}-\left(w_{i}-w_{j}+0.5\right)\right)}^{2}}{w_{i}-w_{j}+0.5}.$$

Consequently, it is necessary to minimize the deviation between the PLPR and its corresponding consistent PLPR. The following programming model is established:

Model 1

(6) $$\begin{array}{l} \min\;F\left(w\right)=\sum_{i=1}^{m}\sum_{j=1}^{m}\frac{{\left(e_{ij}-\left(w_{i}-w_{j}+0.5\right)\right)}^{2}}{w_{i}-w_{j}+0.5}\\ s.t.w_{i}\geq 0,\;\;i=1,2,\ldots ,m,\;\;\;\sum_{i=1}^{n}w_{i}=1 \end{array}.$$

Since Model 1 is a multi-objective programming model, we can solve it by introducing a pair of positive and negative variance variables in the constraint condition, which is

(7) $$\min\;D=\sum_{i=1}^{m}\sum_{j=1,i\neq j}^{m}\left(d_{ij}^{+}+d_{ij}^{-}\right)$$

Model 2

(8) $$s.t.\left\{ \begin{array}{l} \frac{{\left(e_{ij}-\left(w_{i}-w_{j}+0.5\right)\right)}^{2}}{w_{i}-w_{j}+0.5}-d_{ij}^{+}+d_{ij}^{-}=0,\;\;i,j=1,2,\cdots ,m,\;\;i\neq j\\ \sum_{i=1}^{n}w_{i}=1,\;w_{i}\geq 0,\;i=1,2,\ldots ,m\\ d_{ij}^{+},d_{ij}^{-}\geq 0,\;\;i,j=1,2,\cdots ,m,\;\;i\neq j \end{array}\right.$$

where $d_{ij}^{+}$ and $d_{ij}^{-}$ are the positive and negative deviations of objective F(w), respectively.

**Remark** **1.** *Model 2 is called the probabilistic linguistic chi-square method, which possesses many excellent properties, such as good rank-preserving performance, being a simple algorithm, and having complete coordination. Hence, it is very suitable for a therapeutic schedules evaluation problem with uncertainty.*

### 4.2. The PL-ELECTRE Method

In a MADM problem, $A=\left\{a_{i}|i=1,2,\cdots ,m\right\}$ is a set of indicators and $X=\left\{x_{k}|k=1,2,\cdots ,n\right\}$ is a set of alternative schedules. The experts provide the PLTSs $L{\left(p\right)}_{ki}$ for all i=1,2,⋯,m and k=1,2,⋯,n to indicate the judgements on the alternative schedule $x_{k}$ with respect to the indicator $a_{i}$. All PLTSs construct a probabilistic linguistic decision matrix (PLDM) $R={\left(L{\left(p\right)}_{ki}\right)}_{n\times m}$.

#### 4.2.1. Concordance, Discordance, and Indifferent Sets

To efficiently compare the PLTSs, we first introduce the concept of possibility degree of PLTSs [32] as follows: for any two PLTSs $L{\left(p\right)}_{1}$ and $L{\left(p\right)}_{2}$, the possibility degree of $L{\left(p\right)}_{1}$ being greater than or equal to $L{\left(p\right)}_{2}$ is

(9) $$p\left(L{\left(p\right)}_{1}\geq L{\left(p\right)}_{2}\right)=0.5\left(1+\frac{\left(ae{\left(L_{1}\right)}^{-}-e{\left(L_{2}\right)}^{-}\right)+\left(ae{\left(L_{1}\right)}^{+}-e{\left(L_{2}\right)}^{+}\right)}{\left|ae{\left(L_{1}\right)}^{-}-e{\left(L_{2}\right)}^{-}\right|+\left|ae{\left(L_{1}\right)}^{+}-ae{\left(L_{2}\right)}^{+}\right|+ae\left(L_{1}\cap L_{2}\right)}\right)$$

where $L^{-}=\min\left(r^{\left(l\right)}\right)$, $L^{+}=\max\left(r^{\left(l\right)}\right)$ and $ae{\left(L_{1}\right)}^{l}=p_{1}^{l}L_{1}^{l}$, $ae\left(L_{1}\cap L_{2}\right)=\min\left(p_{1}^{l}L_{1}^{l},p_{2}^{l}L_{2}^{l}\right)$, $L_{1}^{l},L_{2}^{l}\in$ $L_{1}\cap L_{2}$, which denotes the area of the intersection between $L{\left(p\right)}_{1}$ and $L{\left(p\right)}_{2}$.

The greater the possibility degree is, the higher the degree of $L{\left(p\right)}_{1}$ being better than $L{\left(p\right)}_{2}$ is Based on the possibility degree of PLTSs, we below establish the concordance set of alternative schedules.

**Definition** **2.** *Let* $R={\left(L{\left(p\right)}_{ki}\right)}_{n\times m}$ *be a PLDM, which manifests the judgements of the alternative schedules in* $X=\left\{x_{k}|k=1,2,\cdots ,n\right\}$ *with respect to the indicators in* $A=\left\{a_{i}|i=1,2,\cdots ,m\right\}$ *. For any two alternative schedules* $x_{q}$ *and* $x_{k}$ *, the probabilistic linguistic concordance set of them can be defined as:*

(10) $$I_{C}=\left\{i|L{\left(p\right)}_{qi}≻L{\left(p\right)}_{ki}\right\},\;\mathrm{for}\;\mathrm{all}\;i=1,2,\cdots ,m$$

*where* $I_{C}$ *is the set of subscripts for all indicators, where* $L{\left(p\right)}_{qi}$ *is better than* $L{\left(p\right)}_{ki}$ *for* q,k∈{k=1,2,⋯,n} *.*

Furthermore, we can divide this concordance set into three categories:

(1) The strong concordance set ${\overset{⌢}{I}}_{C_{qk}}$
  (11) $${\overset{⌢}{I}}_{C_{\mathrm{qk}}}=\left\{i|\alpha <p\left(\left(L{\left(p\right)}_{qi}\right)>\left(L{\left(p\right)}_{ki}\right)\right)\leq 1\right\}$$
(2) The medium concordance set ${\bar{I}}_{C_{qk}}$
  (12) $${\bar{I}}_{C_{qk}}=\left\{i|\beta <p\left(\left(L{\left(p\right)}_{qi}\right)>\left(L{\left(p\right)}_{ki}\right)\right)\leq \alpha \right\}$$
(3) The weak concordance set ${\overset{⌣}{I}}_{C_{qk}}$
  (13) $${\overset{⌣}{I}}_{C_{\mathrm{qk}}}=\left\{i|0.5<p\left(\left(L{\left(p\right)}_{qi}\right)>\left(L{\left(p\right)}_{ki}\right)\right)\leq \beta \right\}\;$$
  where I={i|i=1,2,…,m} denotes a set of subscripts for all indicators, and α and β, which satisfy 0.5<β<α<1, are the boundaries to divide the types of concordance/discordance sets.

Correspondingly, the discordance set of alternative schedules can be proposed as follows:

**Definition** **3.** *Let* $R={\left(L{\left(p\right)}_{ki}\right)}_{n\times m}$ *be a PLDM, which indicates the judgements of the alternative schedules in* $X=\left\{x_{k}|k=1,2,\cdots ,n\right\}$ *with respect to the indicators in* $A=\left\{a_{i}|i=1,2,\cdots ,m\right\}$ *, then the probabilistic linguistic discordance set of two alternative schedules* $x_{q}$ *and* $x_{k}$ *is*

(14) $$I_{D}=\left\{i|L{\left(p\right)}_{qi}≺L{\left(p\right)}_{ki}\right\},\;\mathrm{for}\;\mathrm{all}\;i=1,2,\cdots ,m$$

*where* $I_{D}$ *is the set of subscripts for all indicators, where* $L{\left(p\right)}_{qi}$ *is worse than* $L{\left(p\right)}_{ki}$ *for* q,k∈{1,2,⋯,n} *.*

Three types of the discordance set are presented as:

(1) The strong discordance set ${\overset{⌢}{I}}_{D_{qk}}$
  (15) $${\overset{⌢}{I}}_{D_{qk}}=\left\{i|\alpha <p\left(\left(L{\left(p\right)}_{qi}\right)>\left(L{\left(p\right)}_{ki}\right)\right)\leq 1\right\}\;$$
(2) The medium discordance set ${\bar{I}}_{D_{qk}}$
  (16) $${\bar{I}}_{D_{qk}}=\left\{i|\beta <p\left(\left(L{\left(p\right)}_{qi}\right)>\left(L{\left(p\right)}_{ki}\right)\right)\leq \alpha \right\}\;$$
(3) The weak discordance set ${\overset{⌣}{I}}_{D_{qk}}$
  (17) $${\overset{⌣}{I}}_{D_{qk}{}^{\prime \prime }}=\left\{i|0.5<p\left(\left(L{\left(p\right)}_{qi}\right)>\left(L{\left(p\right)}_{ki}\right)\right)\leq \beta \right\}.$$

In addition to the above concordance and discordance sets, if $p\left(\left(L{\left(p\right)}_{qi}\right)>\left(L{\left(p\right)}_{ki}\right)\right)=0.5$, then $x_{q}$ is said to be indifferent to $x_{k}$ with respect to the indicator $a_{i}$, which can be expressed by the following probabilistic linguistic indifferent set $I_{0_{qk}}$:

(18) $$I_{0_{qk}}=\left\{i|p\left(\left(L{\left(p\right)}_{qi}\right)>\left(L{\left(p\right)}_{ki}\right)\right)=0.5\right\}$$

where $I_{0}$ is the set of subscripts for all indicators, where $L{\left(p\right)}_{qi}$ is indifferent to $L{\left(p\right)}_{ki}$ for q,k∈{1,2,⋯,n}.

#### 4.2.2. Probabilistic Linguistic Concordance and Discordance Indices

Based on the above concordance, discordance, and indifference sets, we further introduce the concordance and discordance index matrices as follows:

**Definition** **4.** *Let*$C={\left(c_{qk}\right)}_{n\times n}$*be the concordance index matrix of the alternative schedules*$x_{q}$*and*$x_{k}$*for all*q,k=1,2,⋯,n, *and the degree to which*$x_{q}$*is superior to*$x_{k}$*(denoted by*$c_{qk}$*) can be calculated by*

(19) $$c_{qk}={\overset{⌢}{W}}_{C}\times \sum_{i\in {\overset{⌢}{I}}_{C_{qk}}}w_{i}+{\bar{W}}_{C}\times \sum_{i\in {\bar{I}}_{C_{qk}}}w_{i}+{\overset{⌣}{W}}_{C}\times \sum_{i\in {\overset{⌣}{I}}_{C_{qk}}}w_{i}+W_{0}\times \sum_{i\in I_{0_{qk}}}w_{i}$$

*where*$w_{i}$*denotes the corresponding weight of the indicator*$a_{i}$*, and*${\overset{⌢}{W}}_{C}$*,*${\bar{W}}_{C}$*,*${\overset{⌣}{W}}_{C}$*, and*$W_{0}$*are the attitude weights which represent the importance degrees of the strong, medium, and weak concordance sets and the indifference set, respectively. Obviously,*$0\leq c_{qk}\leq 1$*.*

Before we introduce the concept of a discordance index matrix, we first present the following definition:

**Definition** **5.** *Let*$L{\left(p\right)}_{1}$*and*$L{\left(p\right)}_{2}$*be two normalized PLTSs with the corresponding weights*$w_{1}$*and*$w_{2}$*, then the weighted distance of the two PLTSs is*

(20) $$d_{w}\left(w_{1}L{\left(p\right)}_{1},w_{2}L{\left(p\right)}_{2}\right)=\sqrt{\sum_{l=1}^{\#L\left(p\right)}\left(w_{1}p_{1}^{\left(l\right)}\cdot w_{2}p_{2}^{\left(l\right)}\cdot {\left|\frac{r_{1}^{\left(l\right)}-r_{2}^{\left(l\right)}}{T}\right|}^{2}\right)}\;$$

*where*#L(p)*is the length of the PLTSs,*T*is the number of linguistic terms in the set*$S=\{s_{g}|g=-\tau ,$⋯,−1,0,1,⋯,τ}*,*$p_{1}^{\left(l\right)}$*and*$r_{1}^{\left(l\right)}$*are respectively the probability and the subscript of the l*-th *linguistic term in*$L{\left(p\right)}_{1}$*, and*$p_{2}^{\left(l\right)}$*and*$r_{2}^{\left(l\right)}$*are respectively the probability and the subscript of the l*-th *linguistic term in*$L{\left(p\right)}_{2}$*.*

**Definition** **6.** *Let* $D={\left(d_{qk}\right)}_{n\times n}$ *be the discordance index matrix of the alternative schedules* $x_{q}$ *and* $x_{k}$ *for all* q,k=1,2,⋯,n *, then the discordance index for* q,k∈{1,2,⋯,n} *that presents the degree of rejecting that* $x_{q}$ *is superior to* $x_{k}$ *(denoted by* $d_{qk}$ *) can be calculated by*

(21) $$d_{qk}=\frac{1}{\underset{i}{\max}\;d_{w}\left(w_{i}L{\left(p\right)}_{qi},w_{i}L{\left(p\right)}_{ki}\right)}\max({\overset{⌢}{W}}_{D}\times \max\left\{\underset{i\in {\overset{⌢}{I}}_{D_{qk}}}{d_{w}}\left(w_{i}L{\left(p\right)}_{qi},w_{i}L{\left(p\right)}_{ki}\right)\right\},\;{\bar{W}}_{D}\times \max\left\{\underset{i\in {\bar{I}}_{D_{qk}}}{d_{w}}\left(w_{i}L{\left(p\right)}_{qi},w_{i}L{\left(p\right)}_{ki}\right)\right\},{\overset{⌣}{W}}_{D}\times \max\left\{\underset{i\in {\overset{⌣}{I}}_{D_{qk}}}{d_{w}}\left(w_{i}L{\left(p\right)}_{qi},w_{i}L{\left(p\right)}_{ki}\right)\right\})$$

*where* $w_{i}L{\left(p\right)}_{qi}=\left\{L_{qi}^{\left(l\right)}\left(w_{i}p_{qi}^{\left(l\right)}\right)|l=1,2,\cdots ,\#L{\left(p\right)}_{qi}\right\}$ *and* $w_{i}L{\left(p\right)}_{ki}=\left\{L_{ki}^{\left(l\right)}\left(w_{i}p_{ki}^{\left(l\right)}\right)|l=1,2,\cdots ,\#L{\left(p\right)}_{ki}\right\}$ *, respectively.*

#### 4.2.3. Ranking Procedure

In this subsection, we first define the concepts of the strong relationship $O_{S}$ and the weak relationship $O_{W}$ by comparing the concordance and discordance indices with concordance and discordance levels.

**Definition** **7.** *Let* $c^{-}$ *,* $c^{0}$ *, and* $c^{*}$ *be three concordance levels with* $0<c^{-}<c^{0}<c^{*}<1$ *and let* $d^{*}$ *and* $d^{0}$ *be two discordance levels with* $0<d^{0}<d^{*}<1$ *, then*

(1) $x_{q}$ *is strongly superior to* $x_{k}$ *(denoted as* $x_{q}O_{S}x_{k}$ *) if*
  (22) $$x_{q}O_{S}x_{k}\Leftrightarrow \left\{ \begin{array}{l} C\left(x_{q},x_{k}\right)\geq C\left(x_{k},x_{q}\right)\\ \left(C\left(x_{q},x_{k}\right)\geq c^{*},D\left(x_{q},x_{k}\right)\leq d^{*}\right)or\;\;\left(C\left(x_{q},x_{k}\right)\geq c^{0},D\left(x_{q},x_{k}\right)\leq d^{0}\right) \end{array}\right.\;$$
(2) $x_{q}$ *is weakly superior to* $x_{k}$ *(denoted as* $x_{q}O_{W}x_{k}$ *) if*
  (23) $$x_{q}O_{W}x_{k}\Leftrightarrow \left\{ \begin{array}{l} C\left(x_{q},x_{k}\right)\geq c^{-}\\ D\left(x_{q},x_{k}\right)\leq d^{*}\\ C\left(x_{q},x_{k}\right)\geq C\left(x_{k},x_{q}\right) \end{array}\right..$$

**Remark** **2.** *The strong relationship can obtain a more precise and stricter ranking procedure than the weak relationship. However, the weak relationship can keep original information which may be lost in the strong relationship.*

**Figure 1:**
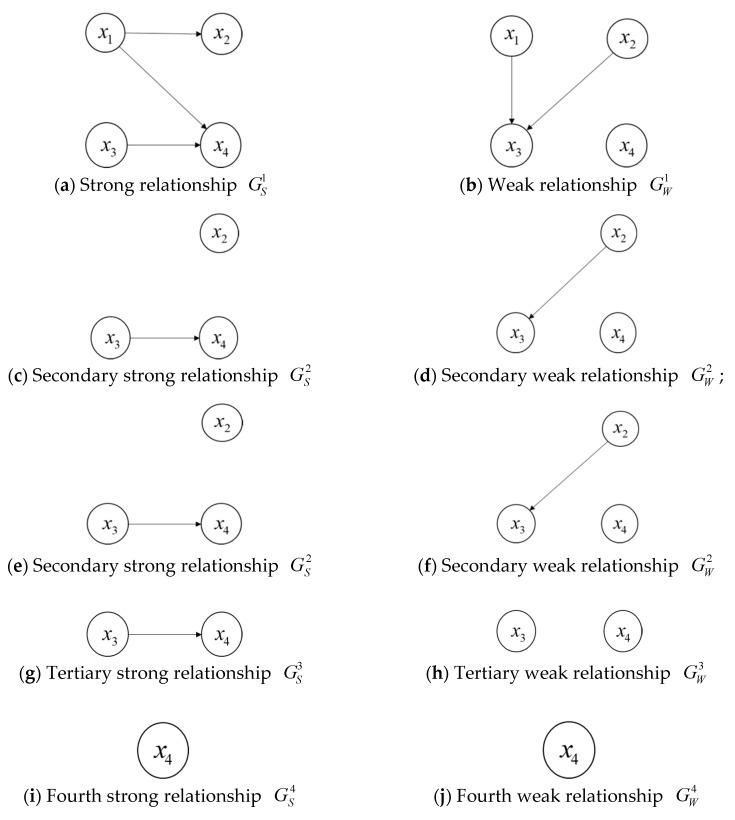
The strong and weak relationships.

If the alternative schedule $x_{q}$ is superior to the alternative schedule $x_{k}$, then we can draw an arrow from $x_{q}$ to $x_{k}$. Based on the above strong relationship and weak relationship, we can respectively construct a strong graph and a weak graph, which can be utilized to obtain the final ranking of alternative schedules according to the topological order [33]. To clearly elaborate the process to derive the final ranking results, we provide an example as follows:

**Example** **1.** *For four alternative schedules*$x_{1}$, $x_{2}$, $x_{3}$*, and*$x_{4}$*, suppose that the strong and weak relationships of them can be shown by the Figure 1a,b:*

**Figure 2:**
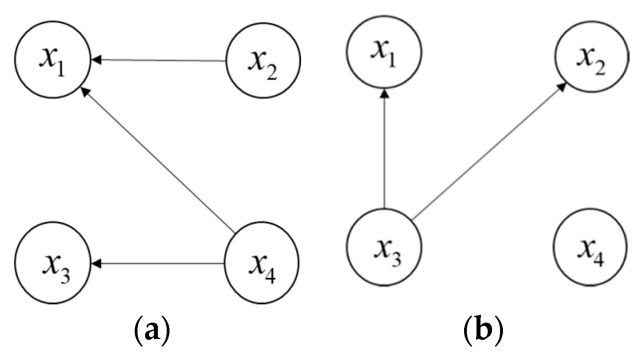
The strong and weak reverse relationships; (**a**) Strong reverse relationship ${\overset{⌢}{G}}_{S}^{1}$; (**b**) Weak reverse relationship ${\overset{⌢}{G}}_{W}^{1}$.

Then, we can conduct the procedure to obtain the ranking of the four alternative schedules:

(1) Firstly, if the degree of an alternative schedule is 0 in the strong relationship or the weak relationship, then the alternative schedule is called a non-dominated alternative schedule. From Figure 1a,b, we can see that $N_{{}_{S}}^{1}=\left\{x_{1},x_{3}\right\}$ is the set of non-dominated alternative schedules in $G_{S}^{1}$ and $N_{W}^{1}=\left\{x_{1},x_{2}\right\}$ is the set of non-dominated alternative schedules in $G_{W}^{1}$. The intersection of $C_{{}_{S}}^{1}$ and $C_{W}^{1}$ (denoted as $N_{}^{1}$) is $N_{}^{1}=\left\{x_{1}\right\}$. Then, the forward ranking $v^{\prime }$ for $x_{1}$ is $v^{\prime }\left(x_{1}\right)=1$.
(2) Next, we can construct the secondary strong relationship $G_{S}^{2}$ and the weak relationship $G_{W}^{2}$ by removing $x_{1}$ in both $G_{S}^{1}$ and $G_{W}^{1}$. In the same way, we can obtain $N_{S}^{2}=\left\{x_{2},x_{3}\right\}$ and $N_{W}^{2}=\left\{x_{2},x_{4}\right\}$, then we record that $N_{}^{2}=\left\{x_{2}\right\}$, and $v^{\prime }\left(x_{2}\right)=2$.
(3) Sequentially, we can construct $G_{S}^{3}$ and $G_{W}^{3}$ by removing $x_{2}$ in both $G_{S}^{2}$ and $G_{W}^{2}$ and obtain $N_{S}^{3}=\left\{x_{3}\right\}$, $N_{W}^{3}=\left\{x_{3},x_{4}\right\}$, then $N_{}^{3}=\left\{x_{3}\right\}$ and $v^{\prime }\left(x_{3}\right)=3$; we can construct $G_{S}^{4}$ and $G_{W}^{4}$ by removing $x_{3}$ in both $G_{S}^{3}$ and $G_{W}^{3}$ and acquire $v^{\prime }\left(x_{k}\right)=\left\{1,2,3,4\right\}$ for k=1,2,3,4.
(4) Furthermore, let all arrows of the directed arcs reverse in $G_{S}^{1}$ and $G_{W}^{1}$ to obtain the reverse graphs ${\overset{⌢}{G}}_{S}^{1}$ and ${\overset{⌢}{G}}_{W}^{1}$ (See Figure 2), then we can obtain $v^{0}\left(x_{k}\right)=\left\{4,3,2,1\right\}$ for k=1,2,3,4 by repeating the above procedures. Later, the reverse ranking $v^{\prime \prime }$ for the four alternative schedules is $v^{\prime \prime }\left(x_{k}\right)$ ={1,2,3,4} for k=1,2,3,4, where $v^{\prime \prime }\left(x_{k}\right)=\max\left(v^{0}\left(x_{k}\right)\right)+1-v^{0}\left(x_{k}\right)$.
(5) Finally, the average ranking $\bar{v}$ can be obtained as $\bar{v}\left(x_{k}\right)=\left\{1,2,3,4\right\}$ for k=1,2,3,4, where $\bar{v}\left(x_{k}\right)=\left[v^{\prime }\left(x_{k}\right)+v^{\prime \prime }\left(x_{k}\right)\right]/2$. The smaller the value of $\bar{v}\left(x_{k}\right)$ is, the greater the alternative schedule is. Therefore, the final ranking of the four alternative schedules is
  (24) $$x_{1}>x_{2}>x_{3}>x_{4}.$$

#### 4.2.4. Algorithm of the Proposed Method

In summary, an algorithm for the PL-ELECTRE II method is presented as follows:

**Step 1.** Invite the experts to give the probabilistic linguistic information to judge each alternative schedule $x_{k}$ with respect to each indicator $a_{i}$, and to compare the indicators in a pair. Correspondingly, we can obtain a PLDM $R={\left(L{\left(p\right)}_{ki}\right)}_{n\times m}$ and a PLPR $B={\left(L{\left(p\right)}_{ij}\right)}_{m\times m}$;

**Step 2.** Derive the weights of all indicators based on Model 2, and calculate the possibility degree between any two alternative schedules with respect to each indicator by Equation (9), denoted as $p(\left(L{\left(p\right)}_{qi}\right)>$ $\left(L{\left(p\right)}_{ki}\right))$, which indicates the possibility degree that an alternative schedule is greater than or equal to another alternative schedule;

**Step 3.** Let the experts provide the attitude weights $W_{0},{\overset{⌢}{W}}_{C},{\bar{W}}_{C},{\overset{⌣}{W}}_{C},{\overset{⌢}{W}}_{D},{\bar{W}}_{D},{\overset{⌣}{W}}_{D}$, the concordance/discordance set division boundaries α and β with 0.5<β<α<1;

**Step 4.** Construct the probabilistic linguistic strong, medium, and weak concordance sets by Equations (11)–(13), the probabilistic linguistic strong, medium, and weak discordance sets by Equations (15)–(17), and the probabilistic linguistic indifferent set by Equation (14);

**Step 5.** Calculate the concordance index matrix by Equation (19) and the discordance index matrix by Equation (21);

**Step 6.** Let the experts determine the concordance levels $c^{-}$, $c^{0}$, and $c^{*}$ with $0<c^{-}<c^{0}<c^{*}<1$ and the discordance levels $d^{*}$ and $d^{0}$ with $0<d^{0}<d^{*}<1$;

**Step 7.** Confirm the strong relationship $O_{S}$ and the weak relationship $O_{W}$ of any two alternative schedules by Equations (22) and (23);

**Step 8.** Draw the strong and weak graphs and obtain the final ranking of alternative schedules.

## 5. Simulation Test

The models in Section 4 possess some parameters, such as the concordance levels ($c^{-},\;c^{0},\;c^{*}$), the discordance levels ($d^{0},\;d^{*}$), and the boundaries to divide the types of concordance/discordance sets (α, β). The authors in [34] pointed out that the ELECTRE II method is very robust when the values of the concordance levels and discordance levels change.

Considering that the values of α and β can affect the construction of the concordance and discordance sets, which may impact the ranking of the alternative schedules, here we endeavor to make some analyses to measure how the parameters α and β influence the ranking results.

**Figure 3:**
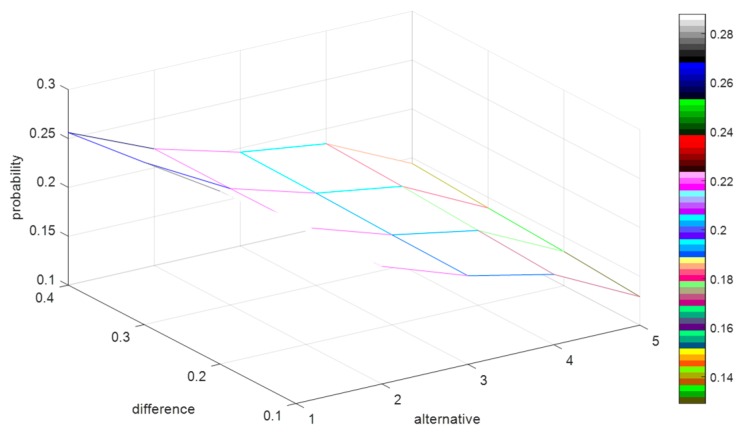
The average probability of each alternative schedule being the optimal solution.

The simulation tests are designed as follows: Randomly generate 1000 decision matrices with five rows and six columns, where the elements of each matrix are presented by PLTSs. Referring to [22], we set $W=\left\{{\overset{⌢}{W}}_{C},{\bar{W}}_{C},{\overset{⌣}{W}}_{C},{\overset{⌢}{W}}_{D},{\bar{W}}_{D},{\overset{⌣}{W}}_{D},W_{0}\right\}=\left\{1,0.9,0.8,1,0.9,0.8,0.7\right\}$, $(c^{-},c^{0},c^{*})=$(0.5,0.6,0.7), and $\left(d^{0},d^{*}\right)=\left(0.6,0.8\right)$. Since the relationship of α and β is defined as 0.5<β<α<1 in Section 4.2, we respectively let α=β+0.1, α=β+0.2, α=β+0.3, and α=β+0.4, and let the values of β increase from 0.5 to 0.9 by adding 0.001 for each step. Then, we record the probability of each alternative schedule being the optimal solution for the 1000 decision matrices. To clearly understand how the difference between α and β impacts on the probability of each alternative schedule being the optimal solution, we calculate the average values of the probabilities for the 1000 decision matrices, which are shown in Figure 3.

**Figure 4:**
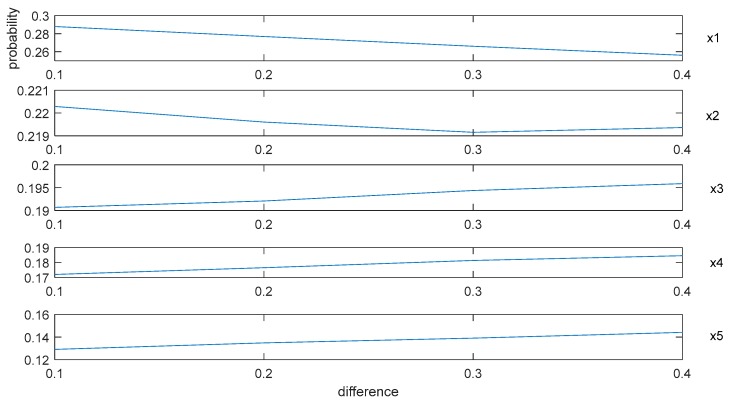
The variation of the difference between *α* and *β*.

In Figure 3, the *x*-axis denotes the alternative schedules, the *y*-axis indicates the value of difference between α and β, and the z-axis represents the probability of each alternative schedule being the optimal solution. To clearly demonstrate the variation of the difference between α and β, we extract the *y*-axis in Figure 3, and then form Figure 4 as:

By Figure 3 and Figure 4, we can summarize some conclusions as follows: (1) when the difference between α and β increases from 0.1 to 0.4, the average probabilities of the alternative schedules $x_{1}$ and $x_{2}$ decrease from 0.29 to 0.26 and from 0.22 to 0.219, respectively, and the average possibility of the alternative schedules $x_{3}$, $x_{4}$, and $x_{5}$ increase from 0.19 to 0.196, from 0.17 to 0.185 and from 0.13 to 0.145, respectively; (2) no matter how the difference between α and β changes, the average probability of $x_{1}$ is the highest. However, it is difficult to explain why the probabilities of $x_{1}$ and $x_{2}$ decrease and the possibilities of $x_{3}$, $x_{4}$, and $x_{5}$ increase based on Figure 3.

**Figure 5:**
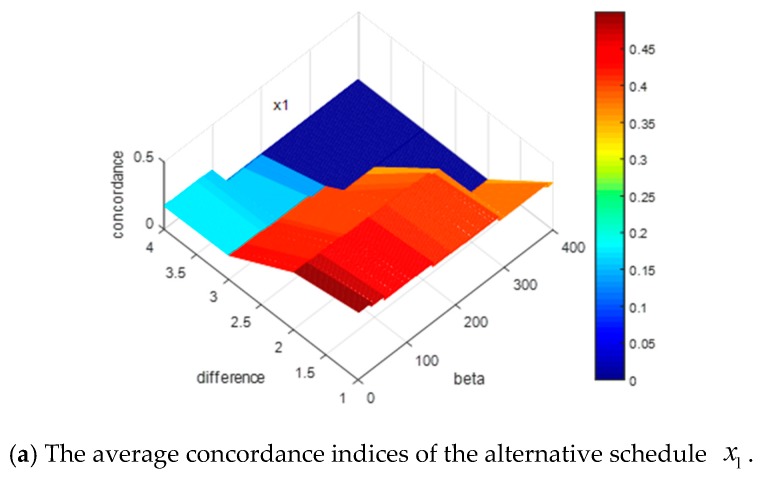

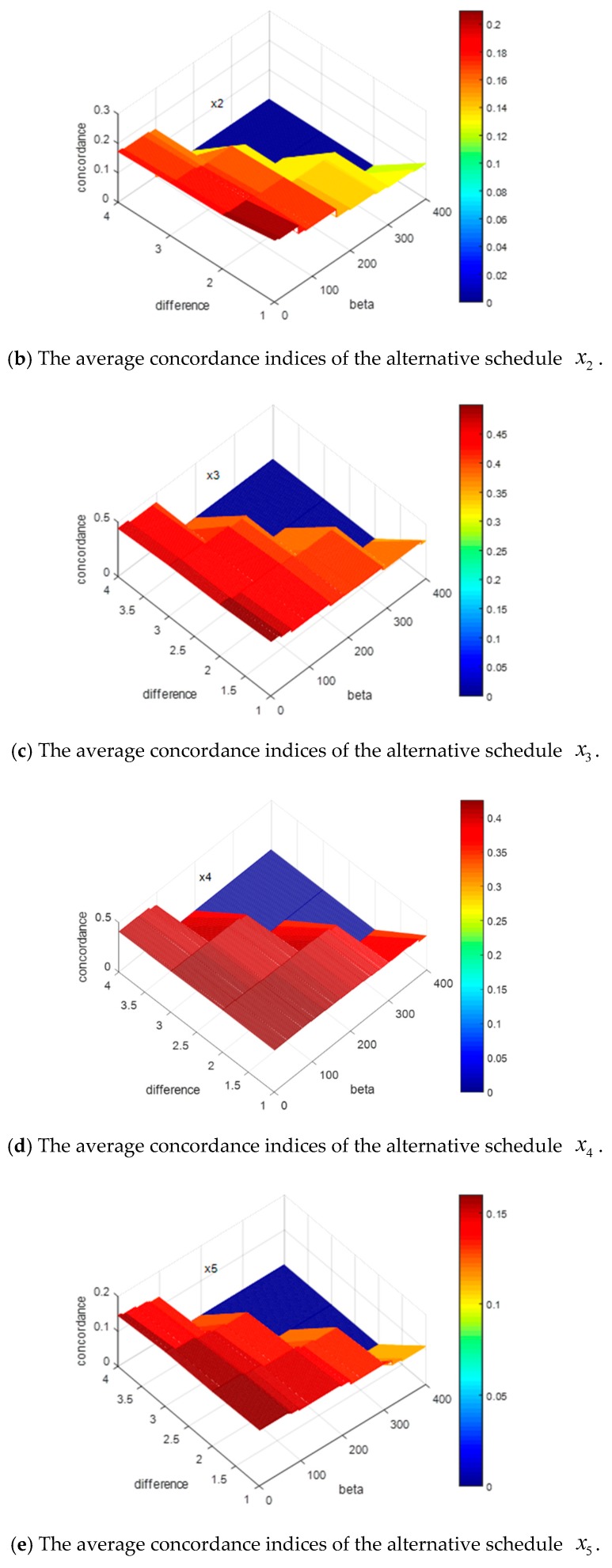
The average concordance indices of alternative schedules.

According to Section 4.2, the construction of the concordance sets and the weight vector of the indicators both are directly related to the concordance indices. Since the weight vectors of the indicators are the same for the 1000 decision matrices and the changes of α and β can affect the construction of the concordance sets, it is essential to discuss how the changes of α and β impact the concordance indices. For convenience, we reduce the dimensions of the matrices by recording the average concordance indices of each alternative schedule for the 1000 decision matrices, where α=β+0.1, α=β+0.2, α=β+0.3, and α=β+0.4. The simulation results are shown in Figure 5.

In Figure 5, the *x*-axis shows the value of β, the *y*-axis denotes the value of difference between α and β, and the z-axis represents the value of the concordance indices for each alternative schedule. Some results can be obtained by the presentation of these figures: (1) when the difference between α and β increases or the value of β increases, all the concordance indices of each alternative schedule decrease; (2) the descending trends of the alternative schedules $x_{1}$ and $x_{2}$ are more obvious than those of the alternative schedules $x_{3}$, $x_{4}$, and $x_{5}$.

In summary, increasing the values of α or β will lead to decreases in the concordance indices. In addition, the descending trends of concordance indices will affect the ranking results of alternative schedules. The more obvious the descending trends of the alternative schedule is, the more possible it is that the alternative schedule will be dominated, which means the ranking of the alternative schedule is more backward. These simulation results can help experts to give some guidance on how to actually assign values to these parameters.

**Remark** **3.** *Since the 1000 decision matrices are generated randomly, the corresponding weighted distances change randomly for the 1000 decision matrices, which causes the discordance indices to change randomly. For such randomness, we assume that the discordance indices have no effect on the results.*

## 6. Case Study

Firstly, for effectively evaluating the proposed method’s performance, we select one patient randomly as our case study from a number of brain-metastasized NSCLC patients in the Sichuan Cancer Hospital and Institute, which is the largest cancer specialist hospital in Southwest China. Then, the evaluation process of five therapeutic schedules for the patient based on the proposed method is represented. At last, some sensitive analyses and comparative analyses are presented to manifest the rationality and stability of the proposed method.

### 6.1. Case Description

A 65-year-old woman was checked out by a Computed Tomography (CT) examination in April 2015, which showed a left upper lung shadow that was found to be a lesion occupying the left upper lung space. The woman underwent a left upper lung sleeve resection in June 2015. After the operation, her postoperative pathology diagnosis revealed low differentiated squamous cell carcinomas in the upper left lung. From 28 June 2015 to 30 September 2015, she endured five rounds of chemotherapy, but there seemed to be no improvement after chemotherapy. In early January 2017, she developed symptoms of fatigue, consciousness disorder, and limb twitching without any obvious cause. In 6 February 2017, she underwent a Brain Magnetic Resonance Imaging (MRI) examination and the result revealed that she had multiple brain metastases.

With the previous treatment process that gave her hardship, the old woman was not willing to undergo surgery again and continue to undergo multiple radiotherapies. Under such complex conditions, it was hard for the attending physician to determine an optimal therapeutic schedule by himself. In the real word, the attending physician gave five optional therapeutic schedules, i.e., “Stereotactic Radiosurgery (SRS) $x_{1}$”, “Whole Brain Radiotherapy (WBRT) $x_{2}$”, “ALK-Targeted Therapies $x_{3}$”, “WBRT-SRS $x_{4}$”, and “Erlotinib or Gefitinib $x_{5}$”, and gathered five experts from Radiotherapy, Neurosurgery, Oncology, Pathology, and Imaging together to consult on an optimal therapeutic schedule for the old woman. Through a long consultation, the experts chose $x_{3}$ as the woman’s therapeutic schedule, and the eutherapeutic of the therapeutic schedule was confirmed by clinical observation.

### 6.2. Evaluation Process

In the proposed method, instead of gathering the five experts together, the attending physician respectively invited five experts to compare all indicators in pairs and provide probabilistic linguistic information to evaluate the five therapeutic schedules with respect to each indicator by referring to Table 1. The PLPR associated with the comparative of the indicators and the PLDM associated with the evaluations of the therapeutic schedules are respectively shown in Table 2 and Table 3.

Based on the above information, the final ranking of the therapeutic schedules can be derived as $x_{3}>x_{5}>x_{4}>x_{1}>x_{2}$ (see Tables S1 and S2 for the specific procedure).

It is easy to find that the therapeutic schedule $x_{3}$ selected by the proposed method is the same as the real schedule chosen by the actual expert consultation. Since the eutherapeutic of the therapeutic schedule $x_{3}$ is confirmed by clinical observation, the optimality of the therapeutic schedule selected by the proposed method can be validated.

### 6.3. The Sensitivity Analyses

#### 6.3.1. Sensitivity Analyses of α and β

**Figure 6:**
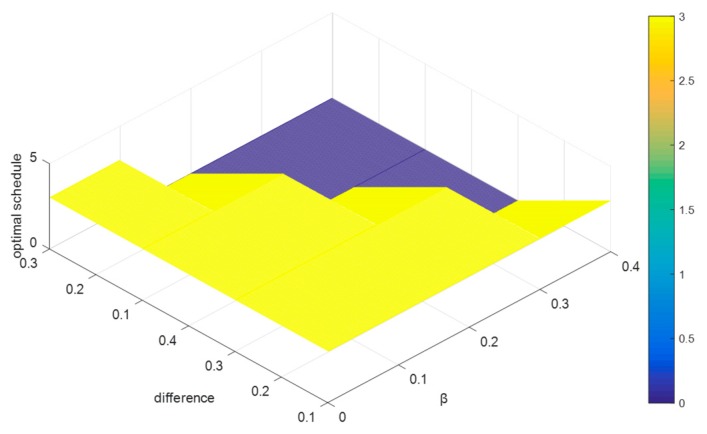
The average rankings of the schedules when *α* and *β* increase.

To testify how the parameters α and β significantly impact the ranking in the case study, we let the values of α and β change as the same way as in Section 5, and record the results of the optimal therapeutic schedule in Figure 6, where $W=\left\{W_{0},{\overset{⌢}{W}}_{C},{\bar{W}}_{C},{\overset{⌣}{W}}_{C},{\overset{⌢}{W}}_{D},{\bar{W}}_{D},{\overset{⌣}{W}}_{D}\right\}=\{0.7,1,$ 0.9,0.8,1,0.9,0.8}, $(c^{-},c^{0},c^{*})=\left(0.5,0.6,0.7\right)$, and $\left(d^{0},d^{*}\right)=\left(0.6,0.8\right)$ and β increase.

From Figure 6, we can conclude that when the difference between α and β increases (or the value of β increases), $x_{3}$ always remains the optimal therapeutic schedule. That is to say, when $W=\left\{W_{0},{\overset{⌢}{W}}_{C},{\bar{W}}_{C},{\overset{⌣}{W}}_{C},{\overset{⌢}{W}}_{D},{\bar{W}}_{D},{\overset{⌣}{W}}_{D}\right\}=\left\{0.7,1,0.9,0.8,1,0.9,0.8\right\}$, $(c^{-},c^{0},c^{*})=\left(0.5,0.6,0.7\right)$, and $\left(d^{0},d^{*}\right)=\left(0.6,0.8\right)$, the changes of α and β have no significant impacts on the optimal selections among the five therapeutic schedules. Since the results in Section 5 show that the concordance indices will decrease with increases in the value of β, we can deduce that the change ranges of the concordance indices do not exceed the levels $(c^{-},c^{0},c^{*})=\left(0.5,0.6,0.7\right)$ and $\left(d^{0},d^{*}\right)=\left(0.6,0.8\right)$.

#### 6.3.2. Sensitivity Analyses of $c^{-}$, $c^{0}$, and $c^{*}$

**Figure 7:**
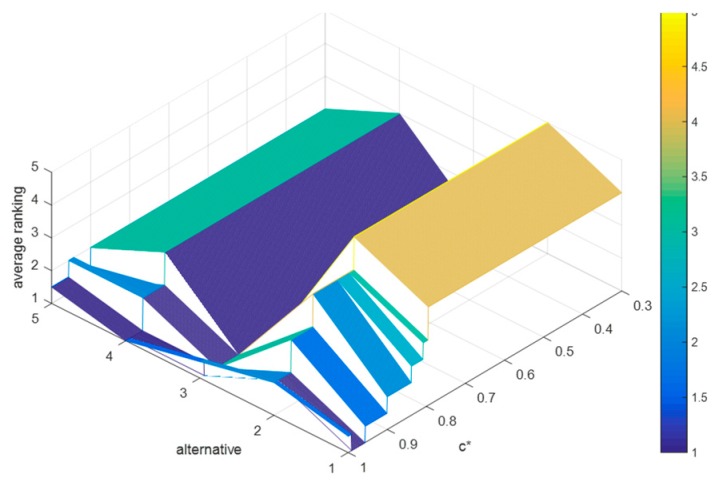
The average rankings of schedules when $c^{*}$ increases from 0.3 to 1.

Suppose that the difference between $c^{-}$ and $c^{0}$ and the difference between $c^{0}$ and $c^{*}$ are both 0.1. Then, we can obtain that $c^{0}=c^{*}-0.1$, $c^{-}=c^{*}-0.2$. Since $0<c^{-}<c^{0}<c^{*}<1$, then the value range of $c^{*}$ is $0.3<c^{*}<1$. Based on which, we let $c^{*}$ increase from 0.3 to 1 in steps with 0.001, and record the average rankings of schedules (as shown in Figure 7), where $W=\left\{W_{0},{\overset{⌢}{W}}_{C},{\bar{W}}_{C},{\overset{⌣}{W}}_{C},{\overset{⌢}{W}}_{D},{\bar{W}}_{D},{\overset{⌣}{W}}_{D}\right\}=$ {0.7,1,0.9,0.8,1,0.9,0.8}, α=0.833, β=0.667, and $\left(d^{0},d^{*}\right)=(0.6,0.8)$.

By Figure 7, we can deduce that: (1) when $c^{*}\in \left(0.3,0.795\right)$, the average rankings of the five schedules remain unchanged, i.e., $x_{3}>x_{5}>x_{4}>x_{1}>x_{2}$ holds for $c^{*}\in \left(0.3,0.795\right)$; (2) when $c^{*}\in \left(0.796,1\right)$, the ranking results are fluctuating frequently; and (3) when $c^{*}\in \left(0.3,0.941\right)$, the optimal therapeutic schedule is still $x_{3}$, but when $c^{*}\in \left(0.942,1\right)$, the optimal therapeutic schedule turns into $x_{4}$.

It is easy to find that when $c^{*}\in \left(0.3,0.795\right)$, the proposed method shows strong robustness in this case, and when $c^{*}\in \left(0.796,1\right)$, some schedules whose concordance indices are less than 0.596 in this case will be no longer superior to other schedules.

#### 6.3.3. Sensitivity Analyses of $d^{0}$, and $d^{*}$

**Figure 8:**
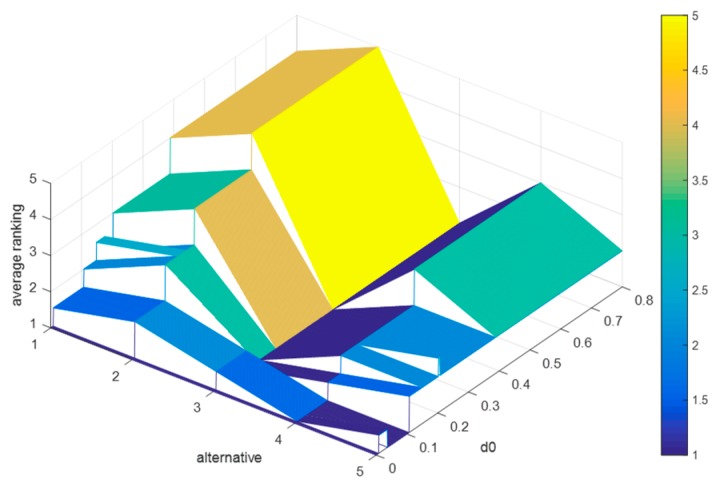
The average rankings of schedules when $d^{0}$ increases from 0 to 0.8.

Suppose that the difference between $d^{0}$ and $d^{*}$ is 0.2, and $d^{0}=d^{*}-0.2$. Since $0<d^{0}<d^{*}<1$, then the value range of $d^{0}$ is $0<d^{0}<0.8$. We let $d^{0}$ increase from 0 to 0.8 in steps of 0.001, and record the average rankings of schedules (as shown in Figure 8), where $(c^{-},c^{0},c^{*})=\left(0.5,0.6,0.7\right)$, $W=\left\{W_{0},{\overset{⌢}{W}}_{C},{\bar{W}}_{C},{\overset{⌣}{W}}_{C},{\overset{⌢}{W}}_{D},{\bar{W}}_{D},{\overset{⌣}{W}}_{D}\right\}=\left\{0.7,1,0.9,0.8,1,0.9,0.8\right\}$, α=0.833, and β=0.667. increases from 0 to 0.8.

By Figure 8, it is obvious that: (1) when $d^{0}\in \left(0,0.389\right)$, the average rankings of the five schedules change markedly; (2) when $d^{0}\in \left(0.39,0.8\right)$, the final ranking is $x_{3}>x_{5}>x_{4}>x_{1}>x_{2}$, which is consistent with the results in the evaluation process; (3) when $d^{0}\in \left(0.109,0.8\right)$, the optimal therapeutic schedule is $x_{3}$, but when $d^{0}\in \left(0,0.107\right)$, the optimal therapeutic schedule is $x_{4}$. Conclusively, when $d^{0}\in \left(0.39,0.8\right)$, the strong relationship and weak relationship between any two schedules do not change, and when $d^{0}\in \left(0,0.389\right)$, some schedules in this case whose discordance indices are greater than 0.389 or 0.589 will no longer be inferior to other schedules, which will lead to a change in the final ranking.

**Remark** **4.** *The above sensitive analyses show that the method is generally stable in this case. The experts can flexibly adjust the values of* $\alpha ,\beta ,c^{-},c^{0},c^{*},d^{0},d^{*}$ *and* $W_{0},{\overset{⌢}{W}}_{C},{\bar{W}}_{C},{\overset{⌣}{W}}_{C},{\overset{⌢}{W}}_{D},{\bar{W}}_{D},{\overset{⌣}{W}}_{D}$ *by referring to the sensitive analyses.*

**Remark** **5.** *It is worth noting that the above analyses are only based on the decision matrices Table 2 and Table 3 in this case and aimed at providing a reference for the experts to assign these parameters’ values in this case study. Hence, they are different from the analyses in Section 5, which are based on 1000 random decision matrices and aimed at verifying the robustness of the proposed method.*

### 6.4. The Comparative Analyses

To prove the reliability of the PL-ELECTRE method, in this subsection, we compare it with the PL-TOPSIS (Technique for Order Preference by Similarity to an Ideal Solution based on PLTSs) method [14] and the PL-AOB (aggregate operator-based with PLTSs) method [27].

#### 6.4.1. Compared with the PL-TOPSIS Method

The PL-TOPSIS method [14] is a MADM method based on a positive ideal solution and a negative ideal solution, which has been verified as a valid decision method in [14]. Based on the decision information of the case study, the process to obtain the ranking of the therapeutic schedules can be listed as follows:

**Step 1.** Based on Table S2, we determine the positive ideal solution (PIS) of the schedules $L_{i}{\left(p\right)}^{+}$ and the negative ideal solution (NIS) of the schedules $L_{i}{\left(p\right)}^{-}$ (The results are shown in Table 4);

**Step 2.** Calculate the closeness coefficients (CI) of each therapeutic schedule by

(25) $$CI(x_{k})=\frac{dev(x_{k},L{\left(p\right)}^{-})}{dev_{\max}(x_{k},L{\left(p\right)}^{-})}-\frac{dev(x_{k},L{\left(p\right)}^{+})}{dev_{\min}(x_{k},L{\left(p\right)}^{+})}$$

where $dev(x_{k},L{\left(p\right)}^{+/-})=\sum_{i=1}^{n}w_{i}dev(L_{ki}(p),L_{i}{\left(p\right)}^{+/-})=\sum_{i=1}^{n}w_{i}\sqrt{\frac{1}{\#L_{ki}(p)}\sum_{l=1}^{\#L\left(p\right)}{\left(p_{ki}^{\left(l\right)}r_{ki}^{\left(l\right)}-{\left(p_{i}^{\left(l\right)}r_{i}^{\left(l\right)}\right)}^{+/-}\right)}^{2}}$ is the deviation degrees between each therapeutic schedule and the PIS (NIS).

**Step 3.** Rank the therapeutic schedules (The results are shown in Table 5).

According to Table 5, the therapeutic schedule $x_{2}$ is the optimal therapeutic schedule, and $x_{3}$ is the worst therapeutic schedule. Based on the above evaluation process and sensitive analyses, we can know that no matter how the values of $\alpha ,\beta ,c^{-},c^{0},c^{*},d^{0},d^{*}$ and $W_{0},{\overset{⌢}{W}}_{C},{\bar{W}}_{C},{\overset{⌣}{W}}_{C},{\overset{⌢}{W}}_{D},{\bar{W}}_{D},{\overset{⌣}{W}}_{D}$ change, the optimal therapeutic schedule obtained by the PL-ELECTRE method is $x_{3}$ or $x_{4}$, and the worst therapeutic schedule is $x_{2}$. By carefully observing the discordance indices matrix, we find that the discordance indices of $x_{2}$ are all more than 0.8, whereas the discordance indices of $x_{3}$ are all less than 0.35. In other words, $x_{2}$ cannot meet the discordance tests in the PL-ELECTRE method, but $x_{3}$ can meet all discordance tests in each indicator. Hence, the results obtained by the PL-ELECTRE method are more reasonable than the ones obtained by the PL-TOPSIS method in this case.

What is more, the main differences between the proposed method and the PL-TOPSIS method are that the PL-TOPSIS method is established based on the positive and negative ideal solutions, which implies the assumption that the relationship between indicators is fully compensatory, where the proposed method can handle indicators with a fully compensatory relationship.

#### 6.4.2. Compared with the PL-AOB Method

The PL-AOB method [27] is a MADM method based on the probabilistic linguistic weighted averaging (PLWA) operator, which is defined as:

(26) $$PLWA\left(L_{1}\left(p\right),L_{2}\left(p\right),\cdots ,L_{n}\left(p\right)\right)\;=\cup_{L_{1}^{\left(l\right)}\in L_{1}\left(p\right)}\left\{w_{1}p_{1}^{\left(l\right)}L_{1}^{\left(l\right)}\right\}⊕\cup_{L_{2}^{\left(l\right)}\in L_{2}\left(p\right)}\left\{w_{2}p_{2}^{\left(l\right)}L_{2}^{\left(l\right)}\right\}⊕\cdots ⊕\cup_{L_{n}^{\left(l\right)}\in L_{n}\left(p\right)}\{w_{n}p_{n}^{\left(l\right)}L_{n}^{\left(l\right)}\}.$$

According to the PLWA operator, we can aggregate the normalized probabilistic linguistic decision matrix and obtain the score of each therapeutic schedule, which can be used to rank therapeutic schedules. All related results are presented in Table 6.

By Table 6, $x_{2}$ is the optimal therapeutic schedule, which is different from the result obtained by the PL-ELECTRE method. One of the reasons is that the PL-AOB method also implies the assumption that the relationship between indicators is fully compensatory; another reason is that aggregating the decision matrix will lose some of the original information. Besides this, by the ranking of the therapeutic schedules in Table 6, there is no difference between $x_{4}$ and $x_{5}$. There is no more information to be utilized to distinguish such an indifferent situation with the PL-AOB method. However, if we solve the case by the PL-ELECTRE method, we can distinguish the two therapeutic schedules by adjusting the values of the parameters, which is considered to be more reasonable and flexible.

## 7. Discussion and Conclusions

The therapeutic schedule problem for brain-metastasized NSCLC has received widespread attention in recent years since millions of people suffer from lung cancer and die from brain-metastasized NSCLC. Based on this situation, the therapeutic schedule evaluation problem for brain metastasized NSCLC is worthy of being discussed, and this paper has established a framework to help to choose the optimal therapeutic schedule for the patients with brain-metastasized NSCLC, including an evaluation indicator system for therapeutic schedules and a probabilistic linguistic ELECTRE II method for the evaluation problem. It can be found that there are five main contributions of this paper:

(1) A novel indicator system is built to evaluate therapeutic schedules for brain-metastasized NSCLC with evidence-based medicine and pharmacoeconomics. The indicator system fully considers the indicators with which patients may be concerned, such as the response of a schedule, the applicability of a schedule, and the evidence of the experts’ evaluations, which guarantees the completeness of the evaluation indicator system;
(2) The decision-making process, which is in line with the form of expert consultation, builds a bridge to link the theoretical decision-making model with practical events. Moreover, compared to traditional expert consultation, it manifests some superiorities:
  (a) Compared to the traditional expert consultation that requires the experts gather together to discuss each therapeutic schedule for obtaining the final result, which will require several hours, the proposed method only needs the experts to give the evaluation values for each therapeutic schedule under each attribute and does not require the experts to be together at the same time or same place;
  (b) Compared to a decision-making process that is invisible and vague in a traditional expert consultation, the decision-making process in the proposed method is visible and clear by using the algorithm of the PL-ELECTRE method;
(3) A PL-ELECTRE method is proposed, which applies probabilistic linguistic information to present the evaluation opinions of the experts, which fits the experts’ expression habits and can keep more of the experts’ information;
(4) The Chi-square method, which possesses a simple algorithm and good rank-preserving performance, is extended into probabilistic linguistic environment to obtain the weight vector of attributes. It provides an efficient, simple, and convenient way to address the individual preference relations of patients.

The paper has also designed some simulation tests to discuss how the parameters in the proposed method impact the rankings of the alternative schedule. Moreover, the paper has provided references for experts about setting parameters.

Furthermore, a case study that helps one patient in Sichuan Cancer Hospital and Institute to evaluate five schedules is developed to verify the rationality of the PL-ELECTRE method. Then, sensitivity analyses based on the different parameters are delivered to prove the stability of the proposed method in the therapeutic schedule evaluation problem. In addition, some comparative analyses by comparing the proposed method with the PL-TOPSIS method and the PL-AOB method were made to investigate the applicability of the PL-ELECTRE method. Nevertheless, the combination of subjective weights and objective weights is a prospective research direction to assist the health service industry, which we will endeavor to address.

## Figures and Tables

**Table 1:** The indicator system.

Object	Attribute	Evaluation Criteria				
	Evaluation Scale	$s_{-2}$	$s_{-1}$	$s_{0}$	$s_{1}$	$s_{2}$
Therapeutic schedule evaluation	Efficacy of response $a_{1}$	Palliative	Minimally effective	Moderately effective	Very effective	Highly effective
	Quality of life $a_{2}$	Poor quality	Low quality	Average quality	Good quality	High quality
	Safety of therapeutic schedule $a_{3}$	Highly toxic	Moderately toxic	Mildly toxic	Occasionally toxic	no toxicity
	Affordability of therapy $a_{4}$	Very expensive	Expensive	Moderately expensive	Inexpensive	Very inexpensive
	Quality of evidence $a_{5}$	Poor quality	Low quality	Average quality	Good quality	High quality
	Consistency of evidence $a_{6}$	Anecdotal evidence only	Inconsistent	May be consistent	Mainly consistent	Highly consistent

**Table 2:** The probabilistic linguistic preference relation (PLPR) of the indicators.

	$a_{1}$	$a_{2}$	$a_{3}$	$a_{4}$	$a_{5}$	$a_{6}$
$a_{1}$	$\left\{s_{0}\left(1\right)\right\}$	$\left\{s_{1}(0.5),s_{2}(0.5)\right\}$	$\left\{s_{2}(0.4),s_{3}(0.6)\right\}$	$\left\{s_{3}(1)\right\}$	$\left\{s_{1}(0.6),s_{2}(0.4)\right\}$	$\left\{s_{1}(0.2),s_{3}(0.8)\right\}$
$a_{2}$	$\left\{s_{-2}(0.5),s_{-1}(0.5)\right\}$	$\left\{s_{0}\left(1\right)\right\}$	$\left\{s_{0}(0.4),s_{1}(0.6)\right\}$	$\left\{s_{1}(0.5),s_{2}(0.5)\right\}$	$\left\{s_{-1}(0.4),s_{0}(0.6)\right\}$	$\left\{s_{0}(0.6),s_{1}(0.3)\right\}$
$a_{3}$	$\left\{s_{-3}(0.6),s_{-2}(0.4)\right\}$	$\left\{s_{-1}(0.6),s_{0}(0.4)\right\}$	$\left\{s_{0}\left(1\right)\right\}$	$\left\{s_{0}(0.6),s_{1}(0.4)\right\}$	$\left\{s_{1}(0.6)\right\}$	$\left\{s_{0}(0.5),s_{1}(0.5)\right\}$
$a_{4}$	$\left\{s_{-3}(1)\right\}$	$\left\{s_{-2}(0.5),s_{-1}(0.5)\right\}$	$\left\{s_{-1}(0.4),s_{0}(0.6)\right\}$	$\left\{s_{0}\left(1\right)\right\}$	$\left\{s_{-2}(0.6),s_{-1}(0.4),\right\}$	$\left\{s_{-2}(0.2),s_{0}(0.8)\right\}$
$a_{5}$	$\left\{s_{-2}(0.4),s_{-1}(0.6)\right\}$	$\left\{s_{0}(0.6),s_{1}(0.4)\right\}$	$\left\{s_{-1}(0.6)\right\}$	$\left\{s_{-3}(0.2),s_{1}(0.8)\right\}$	$\left\{s_{0}\left(1\right)\right\}$	$\left\{s_{1}(0.8)\right\}$
$a_{6}$	$\left\{s_{-3}(0.8),s_{-1}(0.2)\right\}$	$\left\{s_{-1}(0.3),s_{0}(0.6)\right\}$	$\left\{s_{-1}(0.5),s_{0}(0.5)\right\}$	$\left\{s_{0}(0.8),s_{2}(0.2)\right\}$	$\left\{s_{-1}(0.8)\right\}$	$\left\{s_{0}\left(1\right)\right\}$

**Table 3:** The probabilistic linguistic decision matrix (PLDM) of the therapeutic schedules.

	$a_{1}$	$a_{2}$	$a_{3}$	$a_{4}$	$a_{5}$	$a_{6}\;$
$x_{1}$	$\left\{s_{1}(0.4),s_{2}(0.4)\right\}$	$\left\{s_{1}(0.2),s_{2}(0.8)\right\}$	$\left\{s_{-1}(0.4),s_{2}(0.6)\right\}$	$\left\{s_{-2}(0.6),s_{1}(0.4)\right\}$	$\left\{s_{-2}(0.8),s_{-1}(0.2)\right\}$	$\left\{s_{-2}(0.8),s_{1}(0.2)\right\}$
$x_{2}$	$\left\{s_{0}(0.2),s_{2}(0.8)\right\}$	$\left\{s_{1}\left(0.6\right)\right\}$	$\left\{s_{-2}(0.2),s_{-1}(0.8)\right\}$	$\left\{s_{1}(0.2),s_{2}(0.8)\right\}$	$\left\{s_{-1}(0.2),s_{2}(0.8)\right\}$	$\left\{s_{0}(0.2),s_{1}(0.4)\right\}$
$x_{3}$	$\left\{s_{-1}(0.6),s_{2}(0.2)\right\}$	$\left\{s_{2}(0.6),s_{1}(0.4)\right\}$	$\left\{s_{-1}\left(0.2\right),s_{2}\left(0.4\right)\right\}$	$\left\{s_{1}(0.6)\right\}$	$\left\{s_{-2}(0.8),s_{2}\left(0.2\right)\right\}$	$\left\{s_{1}(0.6),s_{2}(0.4)\right\}$
$x_{4}$	$\left\{s_{0}(0.2),s_{1}\left(0.8\right)\right\}$	$\left\{s_{-2}(0.4),s_{2}(0.2)\right\}$	$\left\{s_{2}(0.8)\right\}$	$\left\{s_{1}\left(0.6\right),s_{2}\left(0.4\right)\right\}$	$\left\{s_{-2}(0.6),s_{1}(0.2),\right\}$	$\left\{s_{2}(0.4)\right\}$
$x_{5}$	$\left\{s_{0}(0.4),s_{1}(0.6)\right\}$	$\left\{s_{-2}(0.4),s_{2}(0.2)\right\}$	$\left\{s_{0}(0.2),s_{1}\left(0.6\right)\right\}$	$\left\{s_{-2}(0.2),s_{1}(0.8)\right\}$	$\left\{s_{2}\left(0.6\right)\right\}$	$\left\{s_{-2}(0.2),s_{2}(0.8)\right\}$

**Table 4:** The ideal solutions of the schedules.

	$a_{1}$	$a_{2}$	$a_{3}$	$a_{4}$	$a_{5}$	$a_{6}$
$L_{i}{\left(p\right)}^{+}$	$\left\{s_{0.5},s_{1.6}\right\}$	$\left\{s_{0.4},s_{1.6}\right\}$	$\left\{s_{0},s_{2}\right\}$	$\left\{s_{0.6},s_{1.6}\right\}$	$\left\{s_{0},s_{2}\right\}$	$\left\{s_{0.6},s_{2}\right\}$
$L_{i}{\left(p\right)}^{-}$	$\left\{s_{-0.75},s_{0.5}\right\}$	$\left\{s_{-1.34},s_{0.66}\right\}$	$\left\{s_{-0.4},s_{-0.8}\right\}$	$\left\{s_{-1.2},s_{0.4}\right\}$	$\left\{s_{-1.6},s_{-0.2}\right\}$	$\left\{s_{-1.6},s_{0.2}\right\}$

**Table 5:** The ranking of the schedules with the PL-TOPSIS method.

	$dev^{+}$	$dev^{-}$	$CI_{k}$	**Ranking**
$x_{1}$	0.876	0.67	−1.276	4
$x_{2}$	0.461	0.07	0	1
$x_{3}$	1.061	0.45	−1.880	5
$x_{4}$	0.794	0.718	−1.052	3
$x_{5}$	0.748	0.775	−0.899	2

**Table 6:** The results with the PL-AOB method.

	Aggregated Judgment	Score of Therapeutic Schedule	Ranking
$x_{1}$	$\left\{s_{-0.196},s_{0.753}\right\}$	$s_{0.279}$	4
$x_{2}$	$\left\{s_{-0.400},s_{5.670}\right\}$	$s_{2.635}$	1
$x_{3}$	$\left\{s_{-1.68},s_{5.24}\right\}$	$s_{1.78}$	3
$x_{4}$	$\left\{s_{-2.24},s_{6.51}\right\}$	$s_{2.135}$	2
$x_{5}$	$\left\{s_{-2.14},s_{6.41}\right\}$	$s_{2.135}$	2

## Supplementary Materials

The following are available online at http://www.mdpi.com/1660-4601/15/9/1799/s1, Figure S1: The ordered graphs of the therapeutic schedules in this case, Table S1: The normalized PLPR of the indicators, Table S2: The normalized PLDM of the therapeutic schedules, Table S3: The strong relationship and weak relationship between any two therapeutic schedules, Table S4: The ultimate ranking of the therapeutic schedules.
