# Supplementary material for: Therapeutic Schedule Evaluation for Brain-Metastasized Non-Small Cell Lung Cancer with A Probabilistic Linguistic ELECTRE II Method
Source: Int J Environ Res Public Health. 2018 Aug 21;15(9):1799. doi: 10.3390/ijerph15091799 (PMC6163449; doi:10.3390/ijerph15091799)
Supplement: Supplementary file 1 [file ijerph-15-01799-s001.pdf]

Supplementary materials

1. The procedure of Section 6.2

Step 1. Normalize all PLTSs in PLPR and PLDM according to the rules introduced in Section 2, and obtain the normalized PLPR (as shown in Table 4) and the normalized PLDM (as shown in Table 5);

Table S1. The normalized PLPR of the indicators.

	a_1	a_2	a_3	a_4	a_5	a_6
a_1	$\{s_0(0), s_0(1)\}$	$\{s_1(0.5), s_2(0.5)\}$	$\{s_2(0.4), s_3(0.6)\}$	$\{s_3(0), s_3(1)\}$	$\{s_1(0.6), s_2(0.4)\}$	$\{s_1(0.2), s_3(0.8)\}$
a_2	$\{s_{-2}(0.5), s_{-1}(0.5)\}$	$\{s_0(0), s_0(1)\}$	$\{s_0(0.4), s_1(0.6)\}$	$\{s_1(0.5), s_2(0.5)\}$	$\{s_{-1}(0.4), s_0(0.6)\}$	$\{s_0(0.67), s_1(0.33)\}$
a_3	$\{s_{-3}(0.6), s_{-2}(0.4)\}$	$\{s_{-1}(0.6), s_0(0.4)\}$	$\{s_0(0), s_0(1)\}$	$\{s_0(0.6), s_1(0.4)\}$	$\{s_1(0), s_1(1)\}$	$\{s_0(0.5), s_1(0.5)\}$
a_4	$\{s_{-3}(1), s_{-3}(0)\}$	$\{s_{-2}(0.5), s_{-1}(0.5)\}$	$\{s_{-1}(0.4), s_0(0.6)\}$	$\{s_0(0), s_0(1)\}$	$\{s_{-2}(0.6), s_{-1}(0.4)\}$	$\{s_{-2}(0.2), s_0(0.8)\}$
a_5	$\{s_{-2}(0.4), s_{-1}(0.6)\}$	$\{s_0(0.6), s_1(0.4)\}$	$\{s_{-1}(0), s_{-1}(1)\}$	$\{s_{-3}(0.2), s_1(0.8)\}$	$\{s_0(0), s_0(1)\}$	$\{s_1(0), s_1(1)\}$
a_6	$\{s_{-3}(0.8), s_{-1}(0.2)\}$	$\{s_{-1}(0.33), s_0(0.67)\}$	$\{s_{-1}(0.5), s_0(0.5)\}$	$\{s_0(0.8), s_2(0.2)\}$	$\{s_{-1}(0), s_{-1}(1)\}$	$\{s_0(0), s_0(1)\}$

TableS2. The normalized PLDM of the therapeutic schedules.

	a_1	a_2	a_3	a_4	a_5	a_6
x_1	$\{s_1(0.5), s_2(0.5)\}$	$\{s_1(0.2), s_2(0.8)\}$	$\{s_{-1}(0.4), s_2(0.6)\}$	$\{s_{-2}(0.6), s_1(0.4)\}$	$\{s_{-2}(0.8), s_{-1}(0.2)\}$	$\{s_{-2}(0.8), s_1(0.2)\}$
x_2	$\{s_0(0.2), s_2(0.8)\}$	$\{s_1(0), s_1(1)\}$	$\{s_{-2}(0.2), s_{-1}(0.8)\}$	$\{s_1(0.2), s_2(0.8)\}$	$\{s_{-1}(0.2), s_2(0.8)\}$	$\{s_0(0.33), s_1(0.67)\}$
x_3	$\{s_{-1}(0.75), s_2(0.25)\}$	$\{s_1(0.4), s_2(0.6)\}$	$\{s_{-1}(0.33), s_2(0.67)\}$	$\{s_1(0), s_1(1)\}$	$\{s_{-2}(0.8), s_2(0.2)\}$	$\{s_1(0.6), s_2(0.4)\}$
x_4	$\{s_0(0.2), s_1(0.8)\}$	$\{s_{-2}(0.67), s_2(0.33)\}$	$\{s_2(0), s_2(1)\}$	$\{s_1(0.6), s_2(0.4)\}$	$\{s_{-2}(0.75), s_1(0.25)\}$	$\{s_2(0), s_2(1)\}$
x_5	$\{s_0(0.4), s_1(0.6)\}$	$\{s_{-2}(0.67), s_2(0.33)\}$	$\{s_0(0.25), s_1(0.75)\}$	$\{s_{-2}(0.2), s_1(0.8)\}$	$\{s_2(0), s_2(1)\}$	$\{s_{-2}(0.2), s_2(0.8)\}$

Step 2. By Model 2, the weights of the six indicators are derived as:

$$w = (0.58, 0.08, 0.01, 0.09, 0.12, 0.12)^T \tag{1}$$

Step 3. The experts assign the concordance/discordance set division boundaries α and β as $(\alpha, \beta) = (0.833, 0.667)$, and then they allocate the attitude weights as:

$$W = \{W_0, \widehat{W}_C, \overline{W}_C, \breve{W}_C, \widehat{W}_D, \overline{W}_D, \breve{W}_D\} = \{0.7, 1, 0.9, 0.8, 1, 0.9, 0.8\} \tag{2}$$

Step 4. Calculate $p\left(\left(L(p)_{ij}\right) > \left(L(p)_{kj}\right)\right)$ between any two schedules with respect to each indicator to indicate the possibility degree of an alternative schedule being greater than or equal to another alternative schedule:

$$P=\begin{bmatrix} (0.5\ 0.5\ 0.5\ 0.5\ 0.5\ 0.5) & (0.48\ 0.9\ 0.92\ 0.04\ 0.03\ 0.04) & (0.89\ 0.57\ 0.41\ 0.09\ 0.36\ 0.03) & (0.79\ 0.89\ 0.25\ 0.08\ 0.37\ 0) & (0.82\ 0.89\ 0.53\ 0.17\ 0\ 0.07) \\ (0.52\ 0.1\ 0.08\ 0.96\ 0.97\ 0.96) & (0.5\ 0.5\ 0.5\ 0.5\ 0.5\ 0.5) & (0.89\ 0.2\ 0.06\ 0.9\ 0.93\ 0.23) & (0.9\ 1\ 0\ 0.61\ 1\ 0) & (0.92\ 1\ 0\ 0.94\ 0.29\ 0.3) \\ (0.11\ 0.43\ 0.59\ 0.91\ 0.64\ 0.97) & (0.11\ 0.8\ 0.94\ 0.1\ 0.07\ 0.77) & (0.5\ 0.5\ 0.5\ 0.5\ 0.5\ 0.5) & (0\ 0.89\ 0.29\ 0.36\ 0.51\ 0.38) & (0\ 0.89\ 0.64\ 0.71\ 0.06\ 0.54) \\ (0.21\ 0.11\ 0.75\ 0.92\ 0.63\ 1) & (0.1\ 0\ 1\ 0.39\ 0\ 1) & (1\ 0.11\ 0.71\ 0.64\ 0.49\ 0.62) & (0.5\ 0.5\ 0.5\ 0.5\ 0.5\ 0.5) & (0.6\ 0.5\ 1\ 0.81\ 0\ 0.67) \\ (0.18\ 0.11\ 0.47\ 0.83\ 1\ 0.93) & (0.08\ 0\ 1\ 0.06\ 0.71\ 0.7) & (1\ 0.11\ 0.36\ 0.29\ 0.94\ 0.46) & (0.4\ 0.5\ 0\ 0.19\ 1\ 0.33) & (0.5\ 0.5\ 0.5\ 0.5\ 0.5\ 0.5) \end{bmatrix} \tag{3}$$

Step 5. Construct the types of concordance, discordance indifferent sets based on the matrix P as:

$$I_{\hat{C}}=\begin{bmatrix} - & 4,5,6 & 4,6 & 4,6 & 5,6 \\ 2,3 & - & 3 & 3,6 & 3 \\ 1 & 1,4,5 & - & 1 & 1,5 \\ 2 & 1,2,5 & - & - & 5 \\ 2 & 1,2,4 & - & 3 & - \end{bmatrix},\ I_{\hat{D}}=\begin{bmatrix} - & 2,3 & 1 & 2 & 2 \\ 4,5,6 & - & 1,4,5 & 1,2,5 & 1,2,4 \\ 4,6 & 3 & - & - & - \\ 4,6 & 3,6 & 1 & - & 3 \\ 5,6 & 3 & 1,5 & 5 & - \end{bmatrix}$$

$$I_{\bar{C}}=\begin{bmatrix} - & - & - & 3 & 4 \\ - & - & 2,6 & - & 5,6 \\ - & - & - & 3 & - \\ 1 & - & 2 & - & - \\ 1 & - & 2,4 & 4 & - \end{bmatrix},\ I_{\bar{D}}=\begin{bmatrix} - & - & - & 1 & 1 \\ - & - & - & - & - \\ - & 2,6 & - & 2 & 2,4 \\ 3 & - & 3 & - & 4 \\ 4 & 5,6 & - & - & - \end{bmatrix}$$

$$I_{\breve{C}}=\begin{bmatrix} - & 1 & 3,5 & 5 & - \\ - & - & - & - & - \\ 2 & - & - & 4,6 & - \\ - & 4 & 5 & - & - \\ 3 & - & 3,6 & 1,6 & - \end{bmatrix},\ I_{\breve{D}}=\begin{bmatrix} - & - & 2 & - & 3 \\ 1 & - & - & 4 & - \\ 3,5 & - & - & 5 & 3,6 \\ 5 & - & 4,6 & - & 1,6 \\ - & - & - & - & - \end{bmatrix},\ I_0=\begin{bmatrix} - & - & - & - & - \\ - & - & - & - & - \\ - & - & - & - & - \\ - & - & - & - & 2 \\ - & - & - & 2 & - \end{bmatrix};$$

Step 6. Calculate the probabilistic linguistic concordance indices and construct the concordance index matrix as:

$$C=\begin{bmatrix} - & 0.792 & 0.319 & 0.320 & 0.321 \\ 0.093 & - & 0.191 & 0.134 & 0.233 \\ 0.638 & 0.790 & - & 0.756 & 0.701 \\ 0.596 & 0.849 & 0.168 & - & 0.178 \\ 0.609 & 0.742 & 0.256 & 0.705 & - \end{bmatrix} \tag{4}$$

Step 7. Calculate the weighted distance d_w between any two schedules with respect to each indicator, and construct the weighted distance matrix D_w. Later on, we obtain the probabilistic linguistic discordance indices based on D_w, and then collect all discordance indices to construct the discordance index matrix D:

$$D_w = \begin{bmatrix} - & (0.036\ 0.014\ 0.007\ 0.021\ 0.031\ 0.024) & (0.141\ 0\ 0\ 0\ 0.015\ 0.049) & (0.082\ 0.017\ 0\ 0.033\ 0.011\ 0.011) & (0.082\ 0.017\ 0.002\ 0\ 0.033\ 0.009) \\ - & - & (0.045\ 0.012\ 0.007\ 0.016\ 0.01\ 0.016) & (0.092\ 0.009\ 0.009\ 0\ 0.015\ 0.019) & (0.08\ 0.009\ 0.005\ 0.018\ 0\ 0.021) \\ - & - & - & (0.068\ 0.024\ 0\ 0.011\ 0.006\ 0) & (0.077\ 0.024\ 0.002\ 0\ 0\ 0.024) \\ - & - & - & - & (0\ 0.000\ 0.003\ 0.021\ 0.012\ 0.000) \\ - & - & - & - & - \end{bmatrix} \tag{5}$$

$$D = \begin{bmatrix} - & 0.38 & 1 & 0.9 & 0.9 \\ 0.86 & - & 1 & 1 & 1 \\ 0.35 & 0.32 & - & 0.31 & 0.28 \\ 0.4 & 0.21 & 1 & - & 0.9 \\ 0.41 & 0.24 & 1 & 0.59 & - \end{bmatrix} \tag{6}$$

Step 8. Based on the concordance level $(c^-, c^0, c^*) = (0.5, 0.6, 0.7)$ and the discordance level $(d^0, d^*) = (0.6, 0.8)$ given by the experts, we determine the strong relationship O_S and the weak relationship O_W of the therapeutic schedules. To this end, the strong relationship O_S and the weak relationship O_W can be expressed by Table 6.

Table S3. The strong relationship and weak relationship between any two therapeutic schedules.

	x_1	x_2	x_3	x_4	x_5
x_1	–	O_S			
x_2		–			
x_3	O_S	O_S	–	O_S	O_S
x_4	O_W	O_S		–	
x_5	O_S	O_S		O_S	–

Step 9. According to the topological order, picture the strong and weak graphs of the therapeutic schedules (as shown in Figure 9);

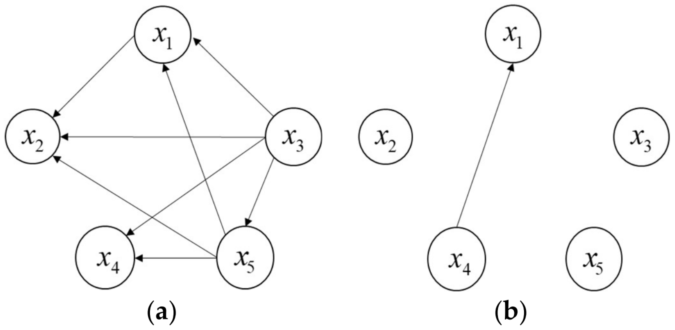

Figure S1. The ordered graphs of the therapeutic schedules in this case. (a) The strong graph; (b) The weak graph.

Step 10. Get the forward ranking $v'(x)$, the reverse ranking $v''(x)$ and the average ranking $\bar{v}(x)$ of the therapeutic schedules (as shown in Table 7), and according to the average ranking $\bar{v}(x)$, the final ranking of the therapeutic schedules can be derived as:

$$x_3 > x_5 > x_4 > x_1 > x_2 \quad (1)$$

Table S4. The ultimate ranking of the therapeutic schedules.

	x_1	x_2	x_3	x_4	x_5
v'	4	5	1	3	2
v''	4	5	1	3	2
$\bar{v}(x)$	4	5	1	3	2
